# The Three-Times Problem: Commentary on Physical Time within Human Time

**DOI:** 10.3389/fpsyg.2023.1130228

**Published:** 2023-06-08

**Authors:** Matt Farr

**Affiliations:** ^1^Department of History and Philosophy of Science, School of the Humanities and Social Sciences, University of Cambridge, Cambridge, United Kingdom; ^2^Wolfson College, University of Cambridge, Cambridge, United Kingdom

**Keywords:** time, passage of time, illusionism, philosophy of time, physical time

## 1. Introduction

Much of our temporal experience is misleading. No doubt this is true in various ways; after all, scientific progress over the centuries has involved giving up ideas that seemed well-motivated by experience. But in the case of time, it has been common to make a very specific set of claims. The “passage” or “flow” of time, and the “presentness” of experience, are often held to be, in some sense, left out of the picture of time described by modern physics. Because of this, passage and presentness have been widely deemed illusory aspects of experience.

In the two feature articles for this volume, Buonomano and Rovelli (2021) and Gruber et al. (2022) focus on what the former call the “two-times problem,” in short, the apparent lack of fit between time as described by physical science and our own temporal experience, where “experience” involves things like memory, anticipation, and perception of change and motion. In this short note I'll make the case that the two-times problem is less serious than it is often made out to be in the specific case of features like “passage” and “presentness“ that are central to the “A-theory” of time — the theory that holds time to be composed of dynamic regions of “past,” “present,” and “future,” and for time to genuinely flow or pass.

My contention is 3-fold: (1) the two-times problem is better understood as a three-times problem: rather than a conflict between “physical” and “manifest” time, what we have in the case of time is differences between the time of physics, the time of experience, and the “folk” concept of time. (2) Understanding the problem in this way helps deflate certain problems about the relationship of these three pictures; the time of experience and the time of physics are less obviously in a problematic conflict than often supposed; and the folk concept of time is what brings in problematic features of time hard to fit with either the time of physics of experience. (3) Understanding the time of experience as independent from the folk concept of time better fits the actual aims of the cognitive neuroscience with respect to the various features of our perception and representation of time.

## 2. The three-times problem

Gruber et al. (2022) use the term “two times problem” to refer to the often-discussed conflict between “physical” and “manifest” time [see also Callender (2017), who introduces and discusses this distinction at length, based on Sellars (1962) famous distinction between the manifest image and scientific image and Eddington's (1928) two-tables problem], wherein physical time lacks certain features central to manifest time, such as passage and presentness.

On the issue of passage, Gruber et al. (2022) note that “the exact mechanism behind this dynamic experience is debatable,” pointing to the diversity of ways of even describing the phenomenon in question, most generally referred to as the “whoosh” of experience, before offering a tentative account in terms of the function of IGUSes [information gathering and utilizing systems, as set out by Hartle (2005) and developed by Callender (2017) and Ismael (2015, 2017)]. Buonomano and Rovelli (2021) see this as a crucial disconnect between physics and neuroscience, suggesting that consilience can be found in explaining the “whoosh” as due to the time asymmetry of thermodynamics. The underlying thought in both cases is that passage/flow is a feature of our temporal experience, but not a feature of the mind-independent world described by physics.

Though there have been various attempts to explain an illusory experience of flow or passage—call this “illusionism”—[notable recent attempts being Paul (2010) and Prosser (2012)], an alternative position has received growing attention. Various authors (e.g., Deng, 2013, 2019; Hoerl, 2014; Farr, 2020; Miller et al., 2020) have motivated the alternative view that the passage of time is not even an illusion, since there is no obvious way in which the flow or passage of time is a feature of our perceptual experience, veridically or illusionary. Instead, Miller et al. (2020) suggest that we can instead think of passage as a kind of “conceptual error” that gives us the false belief that something like flow or passage is a feature of our temporal phenomenology, with Farr (2023) arguing that such concepts are even non-cognitive in nature, that though we describe time in metaphorical terms as flowing like a river, these are not even truth-apt beliefs about our temporal experience. As such, the role of our use of concepts when talking about temporal experience is itself quite distinct from our experience of time itself, and as such it is worth using a 3-fold account of time:

- **Folk Time**. The “folk theory” of time is the way in which we ordinarily describe and conceptualize time.
- **Experienced Time**. The “experience of time” is the multitude of ways in which we perceive various apparently temporal features of the world, such as motion and change.
- **Physical Time**. The “time of physics” is the set of ways in which time is referred to in contemporary physical theory, such as in relativity theory and quantum mechanics.

There is certainly disagreement about what is the “folk theory” of time and good evidence for thinking there's no universally shared folk theory (see Norton, 2021 for a recent overview). However, it is often taken for granted that folk time involves certain features that a central to the “A-theory” of time, such as the primacy of the present moment, the passage of events from future to present and past, and the “flowy,” “dynamic” quality of time. In distinguishing experienced time from folk time, my idea is that we should be careful to distinguish which aspects of ordinary descriptions of time that form the folk theory are themselves aspects of our experience of time, and which are simply due either to false beliefs about our experience of time, or about what time must really be like, or instead some kind of metaphorical mode of describing time.

## 3. The relationship between times, and the aims of cognitive science

In the case of the passage or flow of time, where could a problematic conflict be found between the pictures of time? First we can ask whether physical time really hold time to be “static” in a way that contrasts with temporal experience. It is certainly common to understand relativity theory as portraying time as some kind of static block, mirroring the style of spacetime diagrams used to represent relativistic spacetime. But this is too quick. Many have suggested that relativity theory is perfectly capable of describing the kind of dynamism required to fit with manifest time. And, looking at it from a different perspective, there is a logical problem in holding the traditional four-dimensional block-universe conception of spacetime to be static in a way that contrasts with dynamism, as touched on by Buonomano and Rovelli (2021). They note (following Price, 1996, p. 13) that something ought only be considered as static if unchanging relative to some further variable. A chair is static if it stays still relative to the room surrounding it while the clock on the wall ticks clockwise. But in what sense is a four-dimensional block universe “static,” unless there is an extra, secondary time dimension relative to which it is unchanging? Indeed, the standard response by those that reject the A-theory is that a passageless block universe can (and does) perfectly well give rise to the kinds of temporal experience that we have. In this sense, it is not well-established that physical time excludes the kind of flow or passage common to folk time.

Secondly, we can ask in what ways experienced time involves a notion of flow or passage that could be in conflict with physical time. There are ways in which the brain processes features of the world that are clearly temporal, such as tracking an individual object through a series of changes. And there are ways things appear to us as they change and move that we often refer to as experience of or awareness of time's “flow” or “passage.” And there are the variety of ways in which we invoke the concept of time when recalling one's own memories or projecting forwards to a future event that we are anticipating. Certainly this range of experience gives rise to the idea of time as somehow flowing, and the present being special. It is at this point that many have looked to cognitive science to address deep metaphysical questions about time, such as whether it really passes, or really appears to pass (see Baron et al., 2015 for an overview). However, it is precisely here that I've argued (Farr, 2020) that we risk conflating empirical issues about time perception with a priori issues about the concept of time itself, ultimately conflating metaphysics with cognitive science and misrepresenting the actual aims and subject matter of cognitive science.

Several features of the A-theory, such as passage the privileged present moment, that are out-of-line with the scientific picture of time have widely been thought to stand in need of explanation by the cognitive sciences. However, just because we can describe time in such a way, it does not follow that we experience it as such, and it certainly does not follow that cognitive science is required to explain how illusions of the flow or passage *of time* (as opposed to ordinary moving/changing objects) come about. To focus on our main example of passage, illusionists have searched for various ways in which our brain might falsely represent time as flowing or passing, such as Paul's (2010) suggestion that the “feeling” of passage is a kind of “filling in” effect due to smoothing over temporal snapshots of our local environment, analogous to Wertheimer's famous phi phenomenon, and the suggestion of Gruber et al. (2022) that the sense of flow is due to a representation of “the dynamism of a few temporal experiences from the illusory system, e.g., motion (dynamic movement), dynamic change, and the “feeling of succession” (“pure succession”) of temporality” (p. 9). However, it is important here to note that the sense of “flow” one has from seeing a moving object is at best an analogy for the “flow” of time itself hypothesized by the A-theory, and many have argued that the analogy breaks down in key ways (see Deng, 2013, 2019; Hoerl, 2014; Farr, 2020), motivating the view that such aspects of our cognitive representation of motion and change do not equate to a representation of time as flowing.

Through framing the discrepancies between physical time and folk time as a problem of temporal experience, the metaphysics of time and the experience of time become conflated, together with an implicit pressure on cognitive science to address questions such as “why does time seem to pass.” The trouble is that where there is important work on temporal experience that is relevant, such as change and motion perception, the work itself can be misinterpreted. In the case of motion perception, there are interesting studies on the “flow-like” quality of motion, such as in the famous studies of “motion-blindness” (aka akinetopsia; see Zihl et al., 1983; Zeki, 1991), where subjects lack an ability to sense motion despite seeing objects in sequentially different positions. In such cases there is a reported loss of flow-like elements of motion perception, with Zihl et al. (1983, p. 315) noting the patient's view of a stream of pouring coffee appearing “to be frozen, like a glacier.” It is tempting here to draw the analogy with the idea of time itself appearing as “frozen” as opposed to flowing. However, there are again key differences to keep in mind: coffee can appear frozen through appearing not to continuously change or move over time; but it does not follow that time itself could in any sense appear not to similarly change or move through time.

## 4. In sum

There are many fascinating aspects of our experience of time and our ordinary beliefs and ways of describing time that are incongruous with the properties of time implied by physical theory, as expounded upon by the two feature articles. In this note I've suggested: (1) it is far less clear that the physical and experienced time are in a problematic conflict over any specific property of time; and we must exercise caution when (2) ascribing to “experienced time” certain features central to folk concepts of time that are not clearly aspects of experience, and (3) looking to cognitive science to weigh in on a priori metaphysical issues about the properties of time.

## Author contributions

The author confirms being the sole contributor of this work and has approved it for publication.
